# *In silico* Evolution of Lysis-Lysogeny Strategies Reproduces Observed Lysogeny Propensities in Temperate Bacteriophages

**DOI:** 10.3389/fmicb.2017.01386

**Published:** 2017-07-26

**Authors:** Vaibhhav Sinha, Akshit Goyal, Sine L. Svenningsen, Szabolcs Semsey, Sandeep Krishna

**Affiliations:** ^1^Simons Centre for the Study of Living Machines, National Centre for Biological Sciences-TIFR Bangalore, India; ^2^Manipal University Manipal, India; ^3^Department of Biology, University of Copenhagen Copenhagen, Denmark

**Keywords:** phage-bacteria, mathematical modeling, competition, lysis-lysogeny, *in silico* evolution

## Abstract

Bacteriophages are the most abundant organisms on the planet and both lytic and temperate phages play key roles as shapers of ecosystems and drivers of bacterial evolution. Temperate phages can choose between (i) lysis: exploiting their bacterial hosts by producing multiple phage particles and releasing them by lysing the host cell, and (ii) lysogeny: establishing a potentially mutually beneficial relationship with the host by integrating their chromosome into the host cell's genome. Temperate phages exhibit lysogeny propensities in the curiously narrow range of 5–15%. For some temperate phages, the propensity is further regulated by the multiplicity of infection, such that single infections go predominantly lytic while multiple infections go predominantly lysogenic. We ask whether these observations can be explained by selection pressures in environments where multiple phage variants compete for the same host. Our models of pairwise competition, between phage variants that differ only in their propensity to lysogenize, predict the optimal lysogeny propensity to fall within the experimentally observed range. This prediction is robust to large variation in parameters such as the phage infection rate, burst size, decision rate, as well as bacterial growth rate, and initial phage to bacteria ratio. When we compete phage variants whose lysogeny strategies are allowed to depend upon multiplicity of infection, we find that the optimal strategy is one which switches from full lysis for single infections to full lysogeny for multiple infections. Previous attempts to explain lysogeny propensity have argued for bet-hedging that optimizes the response to fluctuating environmental conditions. Our results suggest that there is an additional selection pressure for lysogeny propensity within phage populations infecting a bacterial host, independent of environmental conditions.

## 1. Introduction

Temperate bacteriophages are crucial players in shaping ecosystems. Recent studies have demonstrated that they play an important role in maintenance of diversity of bacterial communities (Bohannan and Lenski, 2000; Weinbauer and Rassoulzadegan, 2004), in the evolution and competitiveness of bacterial pathogens (Wagner and Waldor, 2002; Davies et al., 2016) and in acquisition of genetic material including antibiotic resistance genes (Balcazar, 2014; Shousa et al., 2015). Temperate bacteriophages have two alternative propagation strategies, lytic and lysogenic growth. During lytic development, the infected bacterial cells produce a large number of phage particles which are released upon host cell lysis. In the lysogenic cycle, most of the phage genes are silenced and the phage genome replicates together with that of the host cell. The lysis-lysogeny decision is regulated by bistable genetic switches (Dodd et al., 1990; Ptashne, 2004; Oppenheim et al., 2005; Avlund et al., 2009a). In the presence of inevitable noise, this typically results in stochastic behavior characterized by a certain lysogeny propensity, i.e., each infected cell has a certain probability to go lysogenic. The lysogeny propensity is determined by the structure of the underlying genetic switch and the way this switch is connected to the intracellular molecular network. A large variety of bistable genetic switches have evolved in nature. Moreover, theoretical studies suggest that such switches can be implemented in many different ways and function in different parameter regimes (Avlund et al., 2009a, 2010). However, observations under laboratory conditions suggest that the lysogeny propensity lies in a narrow range, around 5 to 15%, for a wide range of temperate phage species (Hong et al., 1971; Kourilsky, 1973; Ikeuchi and Kurahashi, 1978; Schubert et al., 2007; Maynard et al., 2010; Broussard et al., 2013). So far, it is not clear why the lysogeny propensities take the values observed. Previous studies (Avlund et al., 2009b; Maslov and Sneppen, 2015) have attempted to explain the observed lysogeny propensity as the result of bet hedging in an uncertain environment. In this scenario, lysogeny propensity reflects the relative likelihood of catastrophes that would destroy free phages or lysogens, and a narrow range of values would require a narrow range of likelihoods for such catastrophes.

Here, we explore an alternate scenario where the lysogeny propensity is determined by competition between phage variants for the same bacterial host. Because bacteriophage DNA mutates at a relatively high rate (Drake, 1991), phage regulatory circuits can be easily tuned for different lysogenization propensities and adapt rapidly to new conditions and co-evolving bacteria (Lapchin and Guillemaud, 2005; Vos et al., 2009). For example, studies in lambda phage and P22 phage have revealed specific mutations in the phage genome—both in regulatory regions as well as coding regions of phage genes—that result in changes in the lysogeny propensity (Kaiser, 1957; Levine, 1957; Kourilsky, 1973; Gottesman and Weisberg, 2004; Schubert et al., 2007). These studies indicate that, in a given phage population, mutants with different lysogenization propensities can emerge and compete with each other for the infection and lysogenization of the host population and, eventually, the optimal propensity can be selected, if it exists. We develop a theoretical framework to address whether lysogeny propensity can be an evolved trait, i.e., whether there is an optimal propensity of lysogeny, in this competitive scenario. We begin our analysis with a simple model where the competing phage variants have a fixed propensity of lysogeny, and extend the model to allow the regulation of the propensity of lysogeny according to the multiplicity of infection. We find that in both scenarios there is, indeed, an optimal strategy, which is not only consistent with the laboratory observations of lysogeny propensities, but also highlights the competitive advantage of phage that can “count,” i.e., phage that can switch from lysis, for single infections, to lysogeny, for multiple infections (Kourilsky, 1973).

## 2. Materials and methods

### 2.1. A model of two phage variants competing for the same bacterial host

Our system consists of two variants of the same phage (with population densities *P*_1_ and *P*_2_) that are identical in all respects except their propensity to go lysogenic. Both compete for a single bacterial strain in our model, but we separately track the density of uninfected bacteria (*B*_0_), the density of bacteria infected by one or the other phage (*B*_1_, *B*_2_) and the density of lysogenized bacteria of each type (*L*_1_, *L*_2_). As we are modeling a well-mixed system, we use ordinary differential equations to represent the dynamics of the bacterial and phage populations:

(1) $$\begin{array}{rcl} \frac{dB_{0}}{dt} & = & \gamma B_{0}\left(1-B_{tot}/K\right)-\eta P_{tot}B_{0} \end{array}$$

(2) $$\begin{array}{rcl} \frac{dB_{i}}{dt} & = & \gamma B_{i}\left(1-B_{tot}/K\right)+\eta P_{i}B_{0}-\delta B_{i} \end{array}$$

(3) $$\begin{array}{rcl} \frac{dL_{i}}{dt} & = & \gamma L_{i}\left(1-B_{tot}/K\right)+f_{i}\delta B_{i} \end{array}$$

(4) $$\begin{array}{rcl} \frac{dP_{i}}{dt} & = & \beta \left(1-f_{i}\right)\delta B_{i}-\eta P_{i}B_{tot} \end{array}$$

where *i* can be 1 or 2, and *B*_*tot*_ and *P*_*tot*_ are the total bacteria and phage densities, respectively. This is a straightforward extension of the model Maynard et al. (2010) use to describe the dynamics of λ phage infecting a population of *E. coli*. The same equations have been used many times previously to model phage-bacteria interaction (Bohannan and Lenski, 2000; Beretta and Kuang, 2001; Weitz and Dushoff, 2007; Maynard et al., 2010; Sneppen et al., 2015). Parameters relating to bacteria are: γ, the bacterial growth rate, and *K*, the carrying capacity, i.e., the maximal total bacterial density the system can sustain. The other parameters are properties of the phages: the “burst size,” β, is the number of phage produced from the lytic infection of one bacterium; the “propensity of lysogeny,” *f*, is the fraction of infected bacteria that go lysogenic; η is the rate constant characterizing infection of bacteria by the phages.

### 2.2. Assumptions

The model assumes that: (i) “super-infection” of lysogens by either phage variant does not change the state of the cell (i.e., lysogens are immune to a subsequent infection), and (ii) “cross-infection” of infected bacteria by the other phage variant also does not change the state of the cell (i.e., the ongoing infection continues, and if the outcome is lytic, only the first type of phage is produced, while if the outcome is lysogeny, only the first type of phage DNA forms a prophage). In both cases, the super-infecting or cross-infecting phage DNA is lost, thus contributing to an effective death rate for the phage [we do not include an additional death rate for phage, assuming that they are stable over the timescales of the dynamics of our model (De Paepe and Taddei, 2006)]. Lysogens are known to be immune to super-infections by phages of their type (Echols, 1972) and we expand this into assumption (i) because the two phage variants are identical in all respects except for their propensity to go lytic or lysogenic, i.e., they share immunity proteins. Note that the model doesn't allow formation of double lysogens. Relaxing assumption (ii) doesn't seem to change our conclusions much, even if we allow cross-infections anytime before the decision (see Supplemental Material Section 1.3). In reality, it is quite likely that a cross-infection only affects the decision if it occurs within a short time window after the first infection, in which case the number of cross infected bacteria is likely to be negligible. Finally, we assume that spontaneous induction of lysogens happens very rarely [≤ 10^−5^/generation (Bæk et al., 2003)] and would not change the final steady-state much, so we ignore this process.

### 2.3. Choice of parameter values

Maynard et al. (2010) obtained bacterial growth curves for a set of experiments where λ phage infected a population of wild type *E. coli* and certain mutants. We fitted a version of the above model with just one phage variant (i.e., Equations 1–4 with *B*_2_, *L*_2_ and *P*_2_ set to zero) to several of these growth curves. This is essentially a repeat of what they have done and we find, as they did, that the model fits well (see Supplemental Figures S1, S2). The range of values we observe for each parameter from this set of fits is shown in column 2 of Table 1. We use these values, along with other experimental observations of parameters, to construct a biologically relevant range of values (column 3 of Table 1). Column 4 of the table shows the much larger ranges of parameter values which we explore in our simulations.

**Table 1:** Parameter values.

**Parameter (units)**	**Range from fitting**	**Biological range**	**Range explored**
γ(*hr*^−1^)	0.93–1.98	0.5–10 (i)	0.2–10^3^
*Kη*(*hr*^−1^)	0.76–13.65	0.45–100 (ii)	0.2–10^3^
β (dimensionless)	21–185	20–1000 (iii)	2–10^4^
δ(*hr*^−1^)	0.2–1.35	0.5–10 (iv)	0.1–10^4^
*f* (dimensionless)	0.00–0.35	0–1	0–1
**Initial conditions**			
*B*_0_(0)/*K* (dimensionless)	10^−5^−10^−3^	10^-9^-1	10^-6^-10^-1^
*P*(0)/*K* (dimensionless)	10^−9^−10^−7^	≥10^-9^	10^-9^-10^-3^

*Column 2 shows the range of parameter values we obtain by fitting the model with a single phage variant to Maynard et al.'s data (2010) of the growth of E. coli under attack by λ phage (see Supplemental Figures S1, S2). Note that we report only the range of the product Kη and not η and K separately. This is because we can always choose the unit in which we measure population density to be the carrying capacity. This corresponds to replacing K by unity and η by Kη (see Section 1.1 in Supplemental Material). Column 3 shows our estimate of a biologically reasonable range of values for each parameter, based on the references cited in the column, which are as follows: (i) Scott et al. (2010), (ii) Ellis and Delbrück (1939); De Paepe and Taddei (2006), (iii) Ellis and Delbrück (1939); De Paepe and Taddei (2006); Wang (2006); Schubert et al. (2007), (iv) Ellis and Delbrück (1939); De Paepe and Taddei (2006); Wang (2006). Column 4 shows the range of parameter values we explore, which is much more extensive than the fits and the biologically reasonable range but is otherwise arbitrary*.

### 2.4. Game-theoretic formulation to find the “optimal” lysogeny propensity

**Figure 1:**
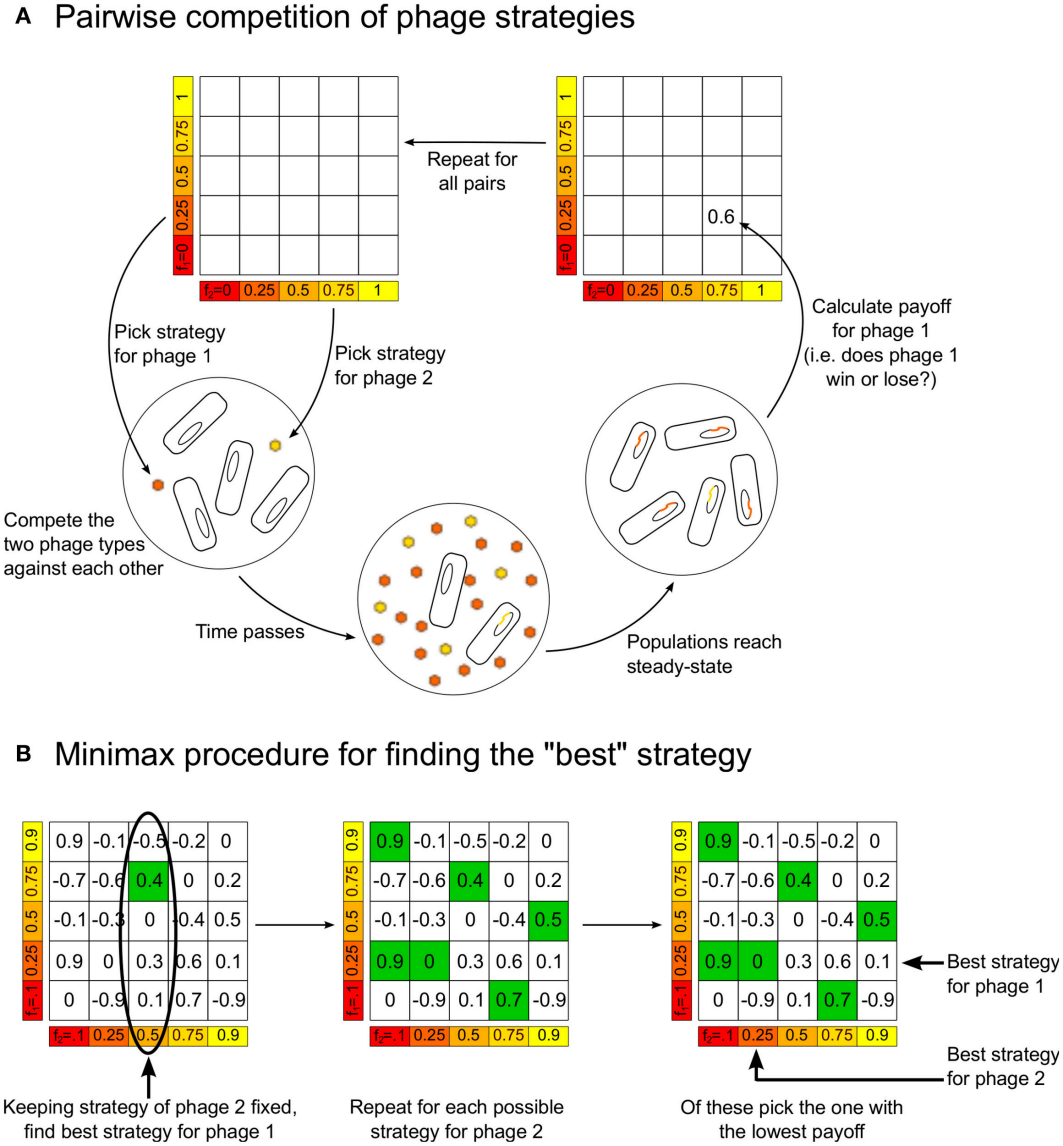
**(A)** Schematic figure showing how the model is used to build up the payoff matrix, by playing each strategy against every other strategy. **(B)** The procedure for determining the “optimal” minimax strategies for the players, given the payoff matrix.

We consider the two phage variants in our model as two players in a game, where the winner is defined to be the variant present in larger numbers as lysogens when the dynamics of Equations (1)–(4) reaches steady-state. This is because, in the steady-state, all phage and bacterial populations are zero except for the lysogens, *L*_1_ and *L*_2_, and sometimes the uninfected bacterial population, *B*_0_. We quantify the magnitude of the win by assigning a score or “payoff” of (*L*_1_ − *L*_2_)/(*L*_1_ + *L*_2_) to phage/player 1 (correspondingly, the payoff for phage/player 2 would be (*L*_2_ − *L*_1_)/(*L*_1_ + *L*_2_); notice that the sum of payoffs is always zero, making this, in the language of game theory, a two-player zero-sum game.) To complete the game-theoretic formulation, we need to specify the “strategies” each player can choose. In the simplest case, the strategies correspond to different possible values of the lysogeny propensity. Thus, we use the terms “phage using strategy *f*” to indicate a phage variant that has lysogeny propensity *f*. Our model thus consists of a game where we compete two players, phage variant 1 using strategy *f*_1_ and phage variant 2 using strategy *f*_2_, where *f*_1_ and *f*_2_ can take all possible values between 0 and 1. In practice, we calculate the payoffs only for *f*_1_ and *f*_2_ taking the values 0, 0.01, 0.02, …, 0.99, 1, which can be arranged in a “payoff matrix” (see Figure 1A). We later include more complex strategies where the lysogeny propensity is not just a fixed number, but a function of the multiplicity of infection (see next section). Note that, in this paper, we do not explore strategies which would allow, for example, changes of burst size. This is because we want to focus on understanding what determines the lysogeny propensities, so we examine competition between phages that are identical in all respects except for their lysogeny propensity.

Given the payoff matrix, it is possible to define the “best” or “optimal” strategy in different ways. We choose to use the notion of a “minimax” strategy. Figure 1B illustrates how to calculate this from a payoff matrix, but the idea is intuitive: Assuming each player plays the best they can, for each strategy player 2 can play there is a maximum payoff that player 1 can obtain by choosing an appropriate strategy. Therefore, player 2 should play the strategy that minimizes this “maximum payoff” for player 1. In the symmetric zero-sum games we are studying, when there is a unique minimax strategy it has the following property: if both players are playing the minimax strategy and one of them changes its strategy, then the other player can always find a strategy that will beat the player who deviated from the minimax strategy. That is, both players playing the minimax strategy is a sort of “equilibrium”—neither player has an incentive to move away from this.

### 2.5. Including multiple infections in the model

We extend the model to allow for multiple infections simply by adding more variables that keep track of the population densities of bacteria infected by more than one phage particle (as before, we don't allow cross-infections). We allow multiple infections at any time before the lysis-lysogeny decision. We further assume that decisions happen with the same rate δ irrespective of the multiplicity of infection (MOI).

(5) $$\begin{array}{rcl} \frac{dB_{0}}{dt} & = & \gamma B_{0}\left(1-B_{tot}/K\right)-\eta P_{tot}B_{0} \end{array}$$

(6) $$\begin{array}{rcl} \frac{dB_{i,1}}{dt} & = & \eta P_{i}B_{0}-\eta P_{i}B_{i,1}-\delta B_{i,1} \end{array}$$

(7) $$\begin{array}{rcl} \frac{dB_{i,m}}{dt} & = & \eta P_{i}B_{i,m-1}-\eta P_{i}B_{i,m}-\delta B_{i,m} \end{array}$$

(8) $$\begin{array}{rcl} \frac{dL_{i}}{dt} & = & \gamma L_{i}\left(1-B_{tot}/K\right)+\delta \overset{\infty }{\underset{m=1}{\sum }}f_{i}\left(m\right)B_{i,m} \end{array}$$

(9) $$\begin{array}{rcl} \frac{dP_{i}}{dt} & = & \beta \delta \overset{\infty }{\underset{m=1}{\sum }}\left(1-f_{i}\left(m\right)\right)B_{i,m}-\eta P_{i}B_{tot}. \end{array}$$

Here *B*_*i,m*_ is the density of cells infected by *m* individuals of the *i*th phage variant. *f*_*i*_(*m*) indicates the lysogeny propensity for cells infected by *m* individuals of the *i*th variant. In practice, in our simulations, we truncate the multiplicity of infection at 3, which is equivalent to assuming that the propensity of lysogeny is the same for all *m* ≥ 3.

In this case, the phage strategy is now specified by a set of three lysogeny propensities: *f*(1) for MOI = 1, *f*(2) for MOI = 2, and *f*(3) for MOI ≥3. Here, the number of different strategies is much greater making an exhaustive enumeration of the payoff matrix computationally intensive. Therefore, we instead implement an iterated “evolutionary” game to find the optimal strategy: We begin with two phage variants whose strategies are chosen randomly. That is, for each phage variant, *f*(1), *f*(2), and *f*(3) are independently and randomly chosen from the interval [0 1]. With these values, we run our model consisting of Equations (5)–(9) until the system reaches steady-state, and determine the winner by counting lysogens. In the next iteration of the game, the winner retains its strategy while the loser is replaced by a new player with a “mutated” version of the winner's strategy, where a random change of upto 6% has been made to the lysogeny propensities: *f*(*m*) → (1 + 0.01*r*_*m*_)*f*(*m*), where *r*_1_, *r*_2_, and *r*_3_ are random integers uniformly chosen from the interval [−3 3]. Then we run Equations (5)–(9) again with these two phage variants, the winner of the previous iteration vs. its mutant. This procedure of replacing the loser is repeated many times, until the winning strategy stops changing.

### 2.6. Simulations

The ordinary differential equation (ODE) systems of our phage-bacteria population in Sections 2.4 and 2.5 were simulated in Python v2.7.12 using the native ‘odeint’ solver, which uses Adams or BDF methods to solve non-stiff and stiff ODE systems respectively. We used a custom python script to systematically test all combinations of fixed lysogenic propensity (Section 2.4). The payoff matrix and the minimax value and associated lysogeny propensity were then calculated using MATLAB. The spatial simulations mentioned in Sections 3.2 and Supplemental Material Section 1.6 were implemented with custom code written in C++. The above code files have been deposited in a public Github repository (https://github.com/vaibhhav/phage_competition_paper_frontiers).

## 3. Results

### 3.1. There exists a non-zero optimal lysogeny propensity when two phage variants compete

We begin by exploring the simplest case where each phage variant is characterized by a fixed propensity of lysogeny. We start the system described by Equations (1)–(4) with a small susceptible bacterial population, at a density *B*_0_(0) well below its carrying capacity, and we introduce the two phage variants in equal, but tiny, amounts *P*_*i*_(0) [Default starting conditions: *P*_*i*_(0)=$10^{-4}B_{0}\left(0\right)$]. As mentioned earlier, the idea is to keep all parameters as identical as possible between the two phage variants so that we can focus on the effect of differences in lysogeny propensity alone. For these initial conditions, the steady state typically ends up having only lysogens of both types, with the total bacterial population at carrying capacity (sometimes, e.g., for particularly small burst sizes, uninfected bacteria may also survive in this steady-state; in extreme cases, where one of the lysogeny propensities is zero, there may only be one type of lysogen surviving at the end.)

**Figure 2:**
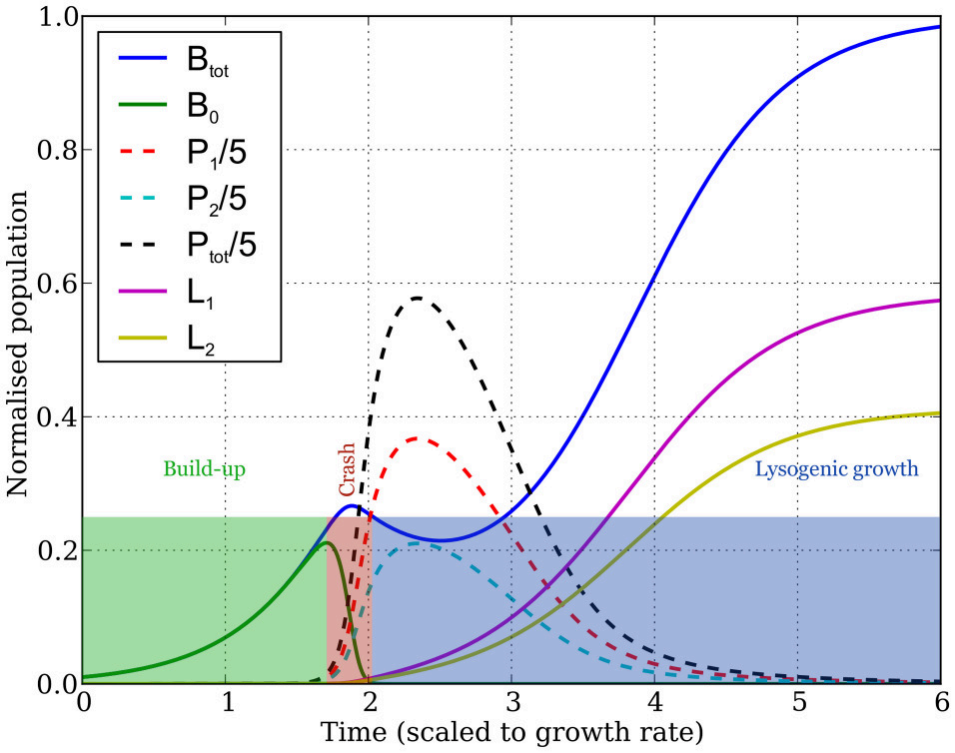
Dynamics of the bacterial and phage populations as a function of time, starting with a tiny amount of the two types of phages infecting a small bacterial population. Parameter values are: (*K* = 1, η = 20, β = 100, γ = 1, δ = 1, *B*_0_(0) = 10^−3^, *P*_*i*_(0) = 10^−7^, *f*_1_ = 0.25, *f*_2_ = 0.29—populations are measured in units of the bacterial carrying capacity, and time in units of the bacterial division time, hence all parameters are dimensionless). The dynamics observed is typical over the range of parameter values shown in column 4 of Table 1, exhibiting three phases as discussed in the main text and as highlighted by the colored rectangles. The *buildup* phase ends, and the *crash phase* begins, at the point the uninfected bacterial population (green curve) reaches its peak. The third phase (*lysogenic growth*) starts when the uninfected bacterial population becomes much smaller than the lysogen populations, leaving the lysogens (yellow and purple curves) to grow until the total bacterial population (blue curve) reaches carrying capacity. The dynamics of phage populations are shown by the dashed lines (red and blue for phages 1 and 2, respectively, and black for the total phage population).

The dynamics typically goes through three distinct stages (Figure 2):

1. The buildup: initially, uninfected bacteria grow exponentially, phages grow even faster (but their numbers still remain low enough that they don't affect the growth of uninfected bacteria much), and lysogen numbers are another order of magnitude smaller.
2. The crash: the phage numbers swell from negligible to significant in a very short time period, after which they infect most uninfected bacteria very quickly (often within one, or less than one, bacterial generation).
3. Lysogenic growth: the phages eventually die out, after which the lysogens that arise from the bacteria infected during the crash (plus the relatively few formed pre-crash), and any remaining uninfected bacteria, then grow until the bacterial population reaches carrying capacity.

**Figure 3:**
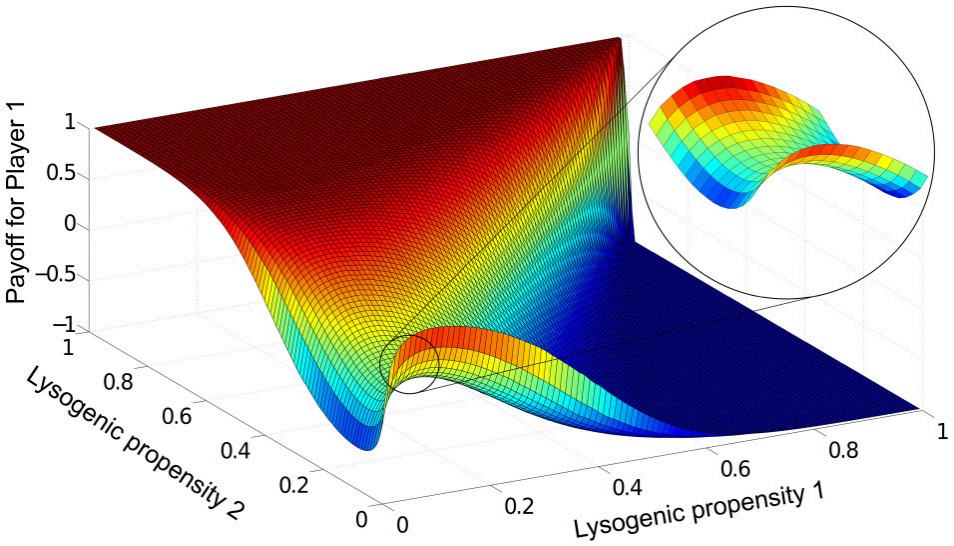
The payoff matrix for Player 1, (*L*_1_ − *L*_2_)/(*L*_1_ + *L*_2_) at steady-state, as a function of *f*_1_ and *f*_2_. Parameters are the same as in Figure 2: *K* = 1, η = 20, β = 100, γ = 1, δ = 1, *B*_0_(0) = 10^−3^, *P*_1_(0)=*P*_2_(0)=10^−7^. The saddle-shaped region (expanded in inset) is characteristic of this particular two-phage game, and the minimax solution in fact lies at the saddle point of the surface. For the range of parameters we explored, we always found the payoff matrix had a unique such saddle point.

In this steady-state, which phage variant dominates the population can be quantified by the “payoff” (*L*_1_ − *L*_2_)/(*L*_1_ + *L*_2_) for phage variant 1, which ranges from +1 when phage variant 1 completely dominates, to -1 when variant 2 completely dominates (see Section 2.4). For a given parameter set, we run Equations (1)–(4) for all possible combinations of lysogeny propensities *f*_1_ and *f*_2_, and record the payoffs for each case in a matrix. Figure 3 shows this payoff matrix for the parameter values used in Figure 2.

To determine from this the optimal lysogeny propensity, we use a game theoretic view of the phage competition (see Section 2.4), treating each run of Equations (1)–(4) as one two-player game, where the players are the two phage variants and the set of strategies for each player are the different possible values of the lysogeny propensity. From the payoff matrix, we can then calculate the optimal “minimax” strategy where each player aims to play what is best for them, *assuming best play by the opponent* (see Figure 1). In Figure 3, this optimal strategy lies at the “saddle point” of the payoff matrix (see inset) and corresponds to a lysogeny propensity *f*_*opt*_ ≈ 0.1. For the entire range of parameters we have examined, we find a unique minimax strategy. This means that if both phage variants have lysogeny propensity *f*_*opt*_, then if either one mutates to have a different lysogeny propensity (whether higher or lower) the other phage variant can always “find” an appropriate lysogeny propensity that will outcompete the phage that mutated away from *f*_*opt*_—thus neither phage type has any incentive to “choose” a strategy other than *f*_*opt*_.

The intuitive reason for the existence of a non-zero optimal lysogeny propensity is that aiming to maximize the number of lysogens in the steady-state puts two opposing “forces” on the lysogeny propensity of a phage variant: first, a higher lysogeny propensity increases the number of lysogens formed per infection during the crash phase; second, a lower lysogeny propensity increases the number of phages available for infecting uninfected bacteria during the crash. It is not surprising, therefore, that there is a non-zero lysogeny propensity that balances these opposing forces in our simple ecosystem. Note that the competition is essential for these two opposing forces to exist.

### 3.2. The optimal lysogeny propensity is very robust to changes in parameter values

**Figure 4:**
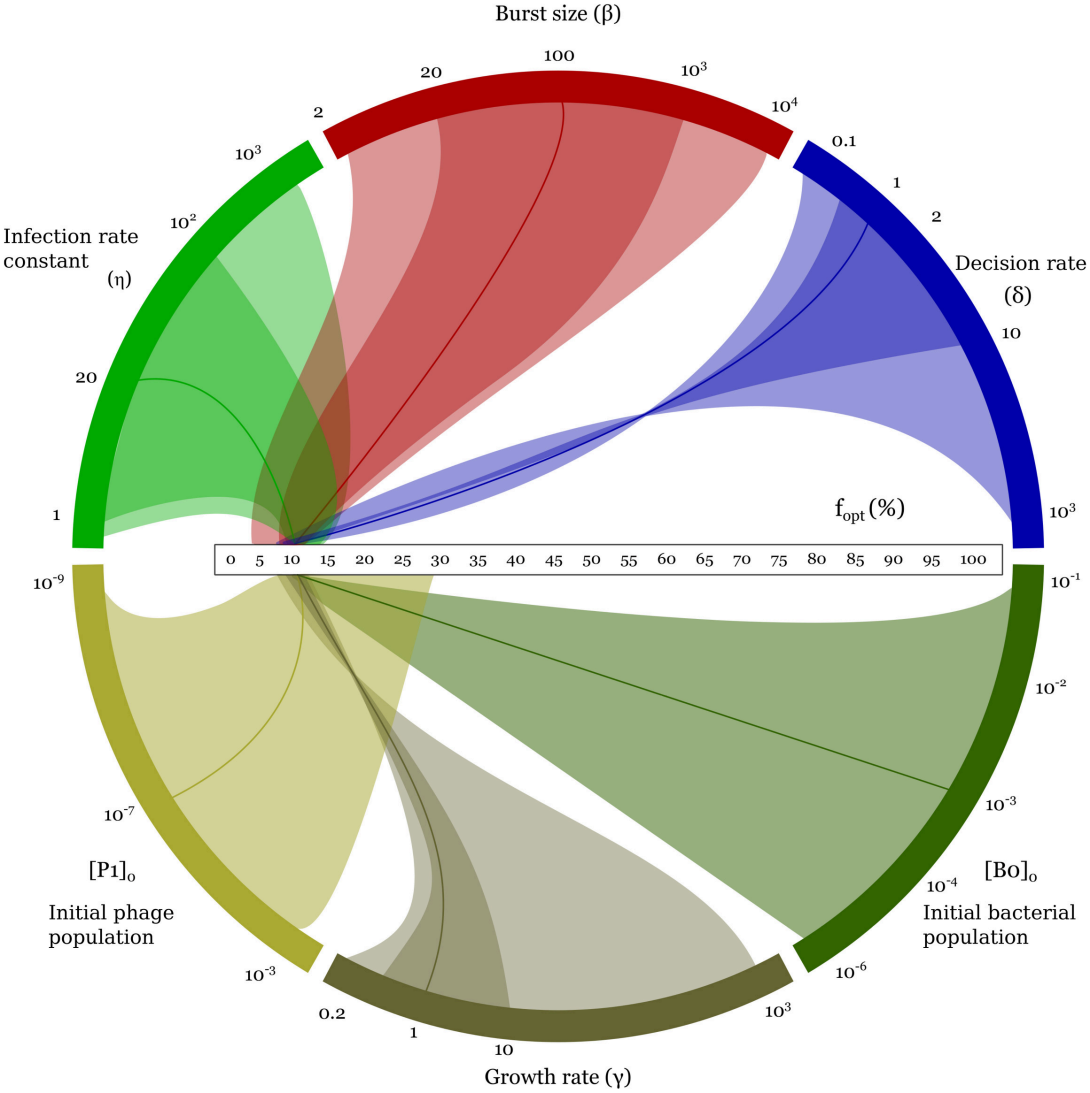
Variation of the optimal (minimax) lysogeny propensity, *f*_*opt*_, as parameters are varied one by one over the range shown in column 4 of Table 1. This figure illustrates the robustness of *f*_*opt*_ over a wide range of parameter values and initial conditions. Every colored arc corresponds to one parameter. The horizontal scale in the middle is the range of possible *f*_*opt*_ values, from 0 to 100%. The solid darker curve connecting each parameter arc to the *f*_*opt*_ scale corresponds to the default parameter set used in Figures 2, 3. The darker and lighter areas connecting the arcs to the *f*_*opt*_ scale mark, respectively, the biologically reasonable range and the full parameter range explored (i.e., columns 3 and 4 of Table 1). The twists indicate decreasing trends for increasing parameter values. For a more conventional representation, see Supplemental Figure S4.

The optimal lysogeny propensity is surprisingly robust to changes in parameter values. Even when the parameters are varied by several orders of magnitude, as shown in Figure 4, we see that *f*_*opt*_ typically lies in the range 5–15%, rising to 30% only when the initial phage population is particularly large. Some trends are visible: *f*_*opt*_ increases with increases in the infection rate constant, η, the burst size, β, the initial phage density, *P*(0), and the initial bacterial density, *B*_0_(0); and decreases with increases in the decision rate, δ, and the bacterial growth rate, γ (see also Supplemental Figure S4). Allowing cross-infections does not affect this result (see Supplemental Material Section 1.3 and Supplemental Figure S3). Incidentally, even when we relax the well-mixed assumption and run a similar game on a spatial 2d-lattice, we observe similar optimal values of lysogeny propensity; see Supplemental Material Section 1.6 and Supplemental Figure S6.

### 3.3. The optimal lysogeny propensity is inversely related to the duration of the buildup phase

Using our observation of the three phases of the dynamics, we can explain this remarkable robustness, and the trends, as follows:

- In the initial phase, because uninfected bacteria grow exponentially *B*_0_ ~ exp(γ*t*), the free phage abundances grow as a double-exponential with rate dependent on their *lytic* propensity: log[*P*_*i*_] ~ (1 − *f*_*i*_) exp(γ*t*). This assumes that decisions happen quickly, i.e., δ is large compared to γ.
- The crash starts when total phage numbers become large enough to make the growth rate of bacteria equal to their rate of infection by phage. That is, if t^*^ denotes the time the crash starts, then $P_{tot}\left(t^{*}\right)\approx \gamma /\eta$.
- Assuming the crash is practically instantaneous (a reasonable assumption for large δ) the remaining uninfected bacteria at this time would become infected with one or other phage variant in proportion to their abundance. That is, a fraction $P_{1}\left(t^{*}\right)/P_{tot}\left(t^{*}\right)$ of bacteria are infected by phage variant 1, and $P_{2}\left(t^{*}\right)/P_{tot}\left(t^{*}\right)$ of bacteria are infected by phage variant 2.
  Thus, at the end of the crash, there are only lysogens and the number of lysogens of type 1 are $f_{1}B_{0}\left(t^{*}\right)P_{1}\left(t^{*}\right)/P_{tot}\left(t^{*}\right)$ and of type 2 are $f_{2}B_{0}\left(t^{*}\right)P_{2}\left(t^{*}\right)/P_{tot}\left(t^{*}\right)$.

The ratio of lysogens $L_{1}/L_{2}=f_{1}P_{1}\left(t^{*}\right)/f_{2}P_{2}\left(t^{*}\right)$ will not change from this point until the steady-state is reached, therefore, the lysogeny propensity that maximizes the payoff for phage/player 1 is the value of *f*_1_ that maximizes $f_{1}P_{1}\left(t^{*}\right)$ (see Supplemental Material Section 1.4 for a more rigorous analysis that calculates the minimax strategy and gives, in Equation (36) in the supplemental material, essentially the same answer). Using the formula in (a) above, this means:

(10) $$\begin{array}{r} f_{opt}\sim e^{-\gamma t^{*}}. \end{array}$$

In other words, the optimal lysogeny propensity depends inversely on t^*^, the time of the crash, or equivalently, the duration of the buildup phase. This already explains some of the trends we observe: for instance, a larger burst size or infectivity would cause the crash to happen earlier, and therefore produces a higher *f*_*opt*_ as we observe in Figure 4.

Under the same assumption of a quick decision, we can combine the arguments (a) and (b) above, to calculate how t^*^ depends on the parameters, finally obtaining (see Supplemental Material Section 1.4):

(11) $$\begin{array}{r} f_{opt}=\frac{1-\frac{1}{\beta }}{1+\ln\frac{\gamma }{2\eta P\left(0\right)}}. \end{array}$$

This explains why the optimal lysogeny propensity is so robust to changes in parameters. Notice that many of the parameters do not even appear in the formula, and the ones that do appear have only a mild effect on the optimal lysogeny propensity because of the logarithm. The burst size is outside the logarithm, but has little effect once it is larger than 10, as it typically is in phages. Supplemental Figure S4 shows that when δ is large, this formula is a good approximation to results obtained from simulations for a large range of parameter values, breaking down only when η, β, *B*_0_(0) and *P*(0) become very large, or when β and γ are small. When δ is brought down to biologically reasonable values, the formula does not predict *f*_*opt*_ as accurately (the simulations differ by Δ*f*_*opt*_ ≈ 0.03) because it ignores the non-negligible time taken to make a decision, but nevertheless it still predicts the robustness and trends well (see Supplemental Figure S4).

### 3.4. Phages that “count” multiplicity of infection compete better

**Figure 5:**
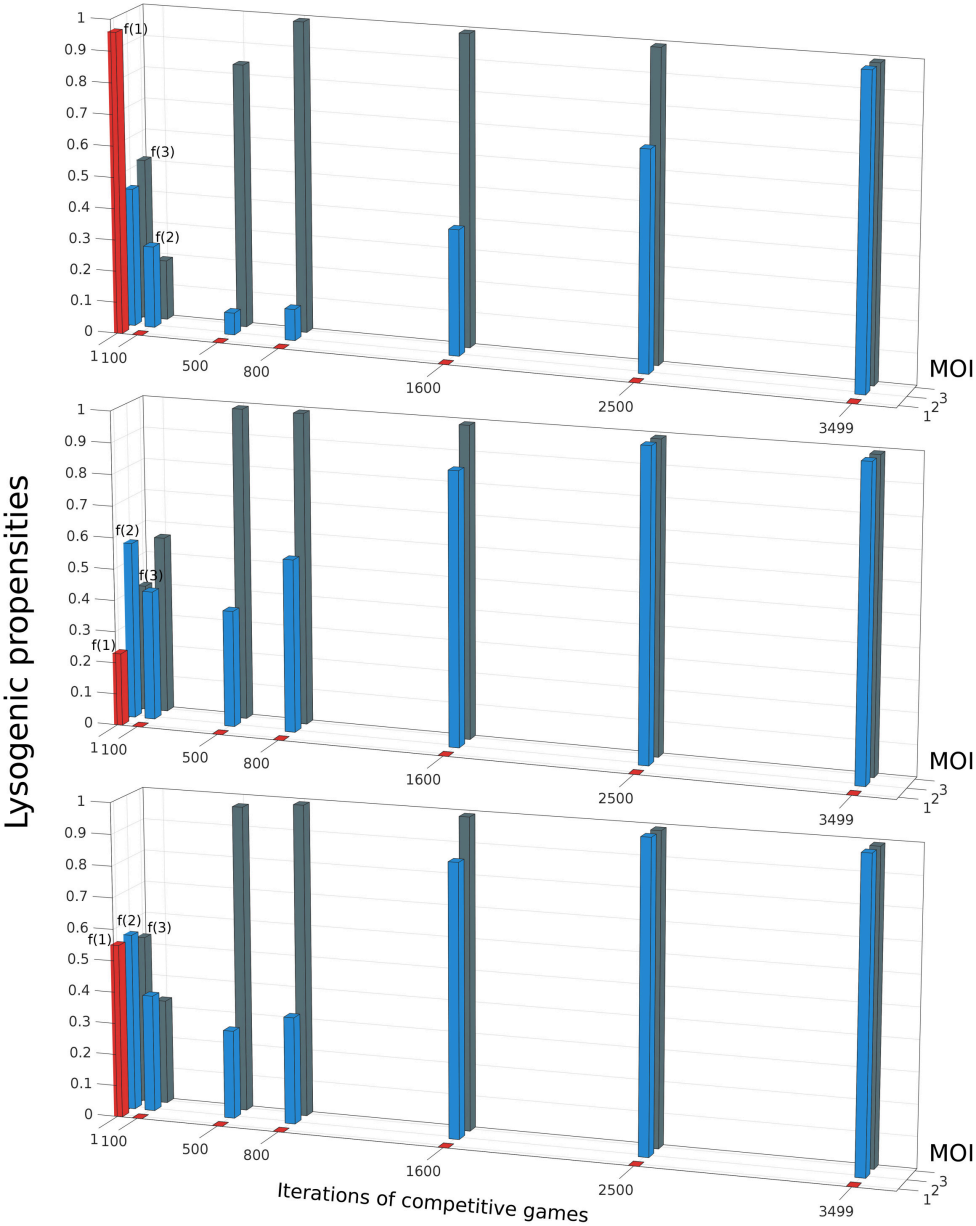
The evolution of the winning strategy *f*(*m*), where *m* is the Multiplicity of Infection (MOI), through iterations of competitive games. The three panels show three independent simulations starting from different initial conditions. In each iteration, if Phage 1 playing strategy *f*_1_(*m*) defeats Phage 2 playing strategy *f*_2_(*m*), then in the next iteration, *f*_2_(*m*) is replaced by *f*_1_(*m*)(1 + 0.01*r*(*m*)), where *r*(*m*) is an integer uniformly distributed in the interval [−3 3]. Despite starting from different initial conditions, all three runs shown share some common trends: *f*(1) rapidly decreases to 0, then *f*(3) rises to 1 and finally *f*(2) also rises to 1. The parameter values and initial conditions used for these runs are the same as in Figures 2, 3. (*K* = 1, η = 20, β = 100, γ = 1, δ = 1, *B*_0_(0) = 10^−3^, $P_{1}\left(0\right)=P_{2}\left(0\right)=10^{-7}$).

Do phages that have a different lysogeny propensity for different multiplicity of infection (MOI) outcompete phages that have a fixed lysogeny propensity that does not depend on MOI? To investigate this, we extend our model to allow multiple infections as described in Section 2.5. A phage “strategy” is now specified by a set of three lysogeny propensities: *f*(1) for MOI = 1, *f*(2) for MOI = 2, and *f*(3) for MOI ≥3 (our model, for simplicity, assumes that the lysogeny propensity is the same for MOI 3 or more). The objective of the games is still the same, to maximize each player's share of lysogens in the steady state. It is computationally rather intensive to exhaustively construct the entire payoff matrix in this case because of the much larger number of possible strategies, so we use a different approach to find the optimal strategy here: Initially, each of the two phage variants starts with random strategies, i.e., randomly chosen values of *f*(1), *f*(2), and *f*(3). With these values, we run our model consisting of Equations (5)–(9) until the system reaches steady-state, and determine the winner by counting lysogens. In the next iteration of the game, the winner retains its strategy while the loser is replaced by a new player with a “mutated” version of the winner's strategy (for details see Section 2.5). These two phage variants now compete again, i.e., we run Equations (5)–(9) once more with these two phage variants, and this procedure of replacing the loser is repeated many times. We find that after many such iterations, the winning strategy, for almost all parameter sets, converges to one where phages always go lytic at MOI 1, and always go lysogenic at MOI 2 or larger. That is, *f*(1) ≈ 0,while *f*(2) ≈ *f*(3) ≈ 1 (see Figure 5). Remarkably, this is very close to what can be inferred from Kourilsky's data: *f*(1) = 0.005 ± 0.05, *f*(2) = 0.7 ± 0.3 and *f*(3) = 0.9 ± 0.1 (Kourilsky, 1973; Avlund et al., 2009b). This was also the optimal strategy we found in exhaustive searches over a restricted strategy space, and we specifically checked that this switch-like strategy outcompetes all phages with fixed lysogeny propensity (see Supplemental Material Section 1.5). Therefore, we believe this strategy is the globally optimal one for most parameter values and initial conditions (very rarely we have observed another switch like strategy, *f*(1) ≈ *f*(2) ≈ 0; *f*(3) ≈ 1, beat this one as shown in Supplemental Figure S5). In the non-well-mixed spatial model we found a similar switch-like strategy was best, except that the switch occurred between MOI = 2 and MOI = 3 (see Supplemental Material Section 1.6 and Supplemental Figure S7).

Our hypothesis for why this MOI-dependent strategy is best amongst the strategies we have examined is that it is able to “detect” the impending crash phase better than other strategies, and respond to it by switching from lysis to lysogeny. Thus, we expect that all other strategies would make more “errors”, i.e., either they would go lysogenic too often in the pre-crash phase or they would go lytic too often during the crash phase. Supplemental Figure S5 provides evidence that this is indeed what typically happens.

Apart from converging to a final state that closely resembles Kourilsky's data, this “evolutionary game,” where the loser is repeatedly replaced by a mutated version of the winner, also highlights certain trends. We observe that, irrespective of the propensity values of the initial random phage strategies, we always see the winning phage's lysogeny propensity for MOI = 1 (i.e., *f*(1)) rapidly decrease in the first few iterations, while its lysogeny propensity for MOI = 2 (i.e., *f*(2)) also decreases a little. After *f*(1) has reached close to zero, in the next iterations, the phage's lysogeny propensity for MOI ≥ 3 (i.e., *f*(3)) rapidly increases while *f*(2) also increases, but more slowly. After *f*(3) has stabilized near 1, *f*(2) continues to increases till it also reaches 1. In other words, the strongest selection pressure appears to act to make phages always go lytic with single infections. The next strongest selection pressure seems to be for the phages to always go lysogenic when the multiplicity of infection is large. Finally, there is a relatively weaker selection pressure pushing double infections to go predominantly lysogenic. This matches the uncertainty of these lysogeny propensities inferred from Kourilsky's data: his data implies that *f*(1) is almost certainly very close to 0, and *f*(3) is very close to 1, while *f*(2) could really lie anywhere between 0.4 and 1 and still yield a reasonable fit to observations (Kourilsky, 1973; Avlund et al., 2009b).

## 4. Discussion

In this work we explore lysis-lysogeny decision strategies in a situation where a small number of temperate bacteriophages attack a large number of susceptible host cells. This is a common situation in experimental conditions (e.g., plaque formation) (Mitarai et al., 2016) and also in natural habitats where phages can be carried to new habitats, or mutations of phages allow infection of new host strains (Weitz et al., 2005). An important feature of such bacteriophage attack is that the growth rate of the phage population largely exceeds the growth rate of the host population. This is because the generation time of the phages is typically comparable to, or a bit shorter than that of the host bacteria, but the number of progeny produced from a single infection (burst size) is in the order of hundreds (De Paepe and Taddei, 2006). Consequently, as shown in Figure 2, invasion of the host population has three distinct phases, which we termed build-up, crash and lysogenic growth. This is true for a very wide range of parameter values, although of course the duration of these phases depends on the parameters.

When operating with fixed propensities of lysogeny, phages need to optimize the strategies for the build-up and crash periods at the same time. However, these strategies are conflicting because the build-up period requires accumulation of free phage particles, while the crash period requires lysogenization of the remaining bacterial population. Our simulations and calculations for competition of phages with different fixed propensities of lysogeny show that there is an optimal propensity, which is very robust to changes in other parameters (e.g., adsorption rate, growth rate, burst size, decision time, initial numbers of phages and bacteria), and falls between 5 and 15% for the majority of parameter sets analyzed. This optimum is independent of the actual implementation of the genetic switch that regulates the decision and of its regulation by intracellular signals. The existence of the optimal propensity suggests that it is an evolved feature of bistable switches regulating the lysis-lysogeny decision. Based on the bet-hedging models (Stewart and Levin, 1984; Veening et al., 2008; Avlund et al., 2009b; Maslov and Sneppen, 2015) and theoretical analyses of a phage competition model (Mittler, 1996), phage variants with higher lysogeny propensities would be favored in certain environments. The existence of such variants has been demonstrated experimentally (Jones and Herskowitz, 1978; Knoll, 1979; Altuvia and Oppenheim, 1986). However, propagation of such phage in laboratory conditions, which are similar to our simulated conditions, would quickly select for mutants that have 5 to 15% lysogeny propensity. Several temperate phages have been reported to choose lysogeny rather than lytic development when large numbers of phages simultaneously attack a bacterial cell (Levine, 1957; Goffart-Roskam, 1965; Kourilsky, 1973) [although there also exist phages, for example, phage *P1*, whose lysogenic frequency is independent of the multiplicity of infection (Rosner, 1972)]. By sensing the multiplicity of infection, phages can identify the onset of the crash period and change their strategy accordingly. The best studied example is bacteriophage λ, which evolved an intricate genetic circuit that regulates the propensities of lysogeny according to environmental cues including the multiplicity of infection. Interestingly, the experimentally observed strategy of λ coincides with the optimal strategy inferred from our extended model (Figure 5).

## Author contributions

SK, SLS, and SS conceived and developed the models; VS, AG, and SK performed the simulations and analysis; VS, AG, SLS, SS, and SK wrote the manuscript.

### Conflict of interest statement

The authors declare that the research was conducted in the absence of any commercial or financial relationships that could be construed as a potential conflict of interest.
