# Supplementary material for: In silico Evolution of Lysis-Lysogeny Strategies Reproduces Observed Lysogeny Propensities in Temperate Bacteriophages
Source: Front Microbiol. 2017 Jul 26;8:1386. doi: 10.3389/fmicb.2017.01386 (PMC5526970; doi:10.3389/fmicb.2017.01386)
Supplement: Supplementary file 1 [file Presentation1.PDF]

1 Supplementary Material

1.1 Non-dimensionalising the model equations

By choosing units in which we measure population densities and time, we can non-dimensionalise the model equations (1) - (4) That is, we define new "rescaled" non-dimensional variables: $b_0 = B_0/K$, $b_i = B_i/K$, $B_{tot} = B_{tot}/K$, $p_i = P_I/K$, $P_{tot} = P_{tot}/K$, $\tilde{t} = \gamma t$. In terms of these variables, the equations (1) - (4) become:

$$\gamma K \frac{db_0}{d\tilde{t}} = \gamma K b_0(1 - KB_{tot}/K) - \eta K^2 P_{tot} b_0 \tag{1}$$
$$\gamma K \frac{db_i}{d\tilde{t}} = \gamma K b_i(1 - KB_{tot}/K) + \eta K^2 p_i b_0 - \delta K b_i \tag{2}$$
$$\gamma K \frac{dl_i}{d\tilde{t}} = \gamma K l_i(1 - KB_{tot}/K) + f_i \delta K b_i \tag{3}$$
$$\gamma K \frac{dp_i}{d\tilde{t}} = \beta(1 - f_i)\delta K b_i - \eta K^2 p_i B_{tot}. \tag{4}$$

We can then write these equations with purely non-dimensional quantities:

$$\frac{db_0}{d\tilde{t}} = b_0(1 - B_{tot}) - \tilde{\eta} P_{tot} b_0 \tag{5}$$
$$\frac{db_i}{d\tilde{t}} = b_i(1 - B_{tot}) + \tilde{\eta} p_i b_0 - \tilde{\delta} b_i \tag{6}$$
$$\frac{dl_i}{d\tilde{t}} = l_i(1 - B_{tot}) + f_i \tilde{\delta} b_i \tag{7}$$
$$\frac{dp_i}{d\tilde{t}} = \beta(1 - f_i)\tilde{\delta} b_i - \tilde{\eta} p_i B_{tot}, \tag{8}$$

where $\tilde{\eta} = \eta K/\gamma$ and $\tilde{\delta} = \delta/\gamma$. Thus, this rescaling of variables corresponds simply to setting $K = 1$ and $\gamma = 1$ in the original equations (1) - (4) (if one only rescales population densities but not time, then it corresponds simply to setting $K = 1$ but not changing γ, and then the remaining parameters all are either dimensionless or have units of 1/time, as in Table 1 in the main text.)

1.2 Fitting to data from Maynard, et al.

The green curves shown in S1 and S2 show OD600 measurements at various time points, from 16 experiments where a population of *E. coli* bacteria was infected (at time zero) by λ phage (data supplied to us by Dr. Markus Covert; the 8 experiments shown in S1 are on *E. coli* K12 wild type and the 8 experiments which are labeled in S2 are on various mutant *E.coli* strains; the same data is used in their paper [Maynard et al., 2010]). We fitted the model (Main text equations (1) - (4)) to this data, minimising $\sum_i \frac{|(B_{tot}(i)-OD(i))|}{OD(i)}$, where $B_{tot}(i)$ and $OD(i)$ are, respectively, the total bacterial population from the model, and the OD600 measurements, at time point i. In addition, the fits were constrained to minimise $\max(|B_{tot}(i) - OD(i)|)$. The resulting fits are shown as blue curves in figures S1 and S2, and the range of fitted parameter values across these 16 fits are shown in each panel, and summarized in Table 1 in the main text.

1.3 Including cross-infections

The model equations can be modified to allow for cross-infections as follows:

$$\dot{B_0} = \gamma B_0(1 - B_{tot}/K) - \eta P_{tot} B_0 \tag{9}$$

$$\dot{B_i} = \eta P_i B_0 - \eta P_j B_i - \delta B_i \tag{10}$$

$$\dot{B_{12}} = \eta(P_1 B_2 + P_2 B_1) - \delta B_{12} \tag{11}$$

$$\dot{L_i} = \gamma L_i(1 - B_{tot}/K) + f_i \delta B_i \tag{12}$$

$$\dot{L_{12}} = \gamma L_{12}(1 - B_{tot}/K) + f_i f_j \delta B_{12} \tag{13}$$

$$\dot{P_i} = \beta\delta(1 - f_i)(B_i + B_{12}) - \eta P_i B_{tot}. \tag{14}$$

Here the two extra variables B_{12} and L_{12} represent, respectively, bacteria infected by both phage variants and double lysogens. The equations assume that phages in infected cells independently make their lysis-lysogeny decision, so double lysogens are formed only when both variants of phage go lysogenic. The payoff now becomes: $Z = (L_1 - L_2)/(L_1 + L_2 + 2L_{12})$. As shown in figure S3, this kind of cross infection doesn't affect the optimal strategy much.

1.4 Analytical derivation of f_{opt} in the limit of very large δ

Simulations suggest that the dynamics of our system, of two phage variants infecting one bacterial species, typically goes through three distinct stages (also see figure 2):

1. The buildup: initially, uninfected bacteria grow exponentially, phages grow even faster (but their numbers still remain low enough that they don't affect the growth of uninfected bacteria much), and lysogen numbers are another order of magnitude smaller.

2. The crash: the phage numbers swell from insignificant to large in a very short time period, after which they infect all, or almost all, uninfected bacteria very quickly (often within one, or less than one, bacterial generation).

3. Lysogenic growth: the phages eventually die out, after which the lysogens that arise from the bacteria infected during the crash (plus the relatively few formed pre-crash) and any remaining uninfected bacteria then grow until the bacterial population reaches carrying capacity.

We will use the above observations to motivate various approximations below that allow us to study the system analytically.

First, we need an estimate of the number of infected bacteria of each type right after the crash. Let t^* be the time at which the crash begins. If $B_0(t^*)$ and $P_i(t^*)$ are the phage and bacterial numbers at that time, then assuming the crash is fast we can take the number of infected bacteria of type i to be $B_0(t^*)P_i(t^*)/P_{tot}(t^*)$. The number of lysogens of type

i produced from these will be $f_i B_0(t^*) P_i(t^*)/P_{tot}(t^*)$. Thus, soon after the crash there will $L_i = f_i B_0(t^*) P_i(t^*)/P_{tot}(t^*)$ lysogens of each type, assuming that the number of lysogens formed before the crash are negligible and the number of bacteria left uninfected is also negligible. Notice that the ratio of lysogen numbers L_1/L_2 is preserved as the lysogens now grow and divide until the total bacterial population carrying capacity. Thus, the ratio of lysogens when the simulations reach steady-state is given by:

$$\frac{L_1}{L_2} = \frac{f_1 P_1(t^*)}{f_2 P_2(t^*)} \tag{15}$$

In order to calculate this ratio analytically, we consider the special case where infected bacteria lyse or turn into lysogens very quickly. This corresponds to the limit $\delta \to \infty$, when the equations reduce to:

$$\frac{dB_0}{dt} = \gamma B_0(1 - B_{tot}) - \eta P_{tot} B_0 \tag{16}$$

$$\frac{dL_i}{dt} = \gamma L_i(1 - B_{tot}) + f_i \eta P_i B_0 \tag{17}$$

$$\frac{dP_i}{dt} = \beta(1 - f_i)\eta P_i B_0 - \eta P_i B_0 \tag{18}$$

To model the buildup, we will assume that uninfected bacteria grow exponentially, $B_0(t) \approx B_0(0)e^{\gamma t}$, i.e., $B_0 \approx B_{tot} \ll 1$ and $P_{tot} \ll \gamma/\eta$. In that case, the dynamics of each phage proceeds independently of the other according to the equations:

$$\frac{dP_i}{dt} = \beta_i' \eta P_i B_0 \tag{19}$$

$$\Rightarrow \frac{dP_i}{dt} = (\beta(1 - f_i) - 1)\eta P_i B_0(0)e^{\gamma t} \tag{20}$$

$$\Rightarrow P_i(t) = P_i(0)e^{\frac{\eta \beta_i' B_0(0)}{\gamma}(e^{\gamma t} - 1)} \tag{21}$$

where $\beta_i' = \beta(1 - f_i) - 1$.

We will assume that this expression is a good approximation to the phage population right

until the end of the buildup phase and where the crash begins, $t = t^*$. At this point, the uninfected bacterial population reaches its maximum, i.e., $\frac{dB_0}{dt}(t^*) = 0$, or:

$$\eta P_{tot}(t^*) \approx \gamma \tag{22}$$

$$\Rightarrow \eta P_1(0) e^{\frac{\eta \beta_1' B_0(0)}{\gamma}(e^{\gamma t^*}-1)} + \eta P_2(0) e^{\frac{\eta \beta_2' B_0(0)}{\gamma}(e^{\gamma t^*}-1)} = \gamma \tag{23}$$

Note that t^* is a function of f_1 and f_2. We will use this equation to calculate the two partial derivatives: $\partial t^* / \partial f_{1,2}$. These will come in use later when calculating the minimax strategy:

$$P_1(0) e^{\frac{\eta \beta_1' B_0(0)}{\gamma}(e^{\gamma t^*}-1)} \frac{\eta B_0(0)}{\gamma} \left[-\beta(e^{\gamma t^*} - 1) + \beta_1' \gamma e^{\gamma t^*} \frac{\partial t^*}{\partial f_1} \right] + P_2(0) e^{\frac{\eta \beta_2' B_0(0)}{\gamma}(e^{\gamma t^*}-1)} \eta \beta_2' B_0(0) e^{\gamma t^*} \frac{\partial t^*}{\partial f_1} = 0 \tag{24}$$

$$\Rightarrow \frac{\partial t^*}{\partial f_1} = \frac{P_1(t^*) \beta (e^{\gamma t^*} - 1)}{\gamma e^{\gamma t^*} (P_1(t^*) \beta_1' + P_2(t^*) \beta_2')} \tag{25}$$

and

$$P_1(0) e^{\frac{\eta \beta_1' B_0(0)}{\gamma}(e^{\gamma t^*}-1)} \eta \beta_1' B_0(0) e^{\gamma t^*} \frac{\partial t^*}{\partial f_2} + P_2(0) e^{\frac{\eta \beta_2' B_0(0)}{\gamma}(e^{\gamma t^*}-1)} \frac{\eta B_0(0)}{\gamma} \left[-\beta(e^{\gamma t^*} - 1) + \beta_2' \gamma e^{\gamma t^*} \frac{\partial t^*}{\partial f_2} \right] = 0 \tag{26}$$

$$\Rightarrow \frac{\partial t^*}{\partial f_2} = \frac{P_2(t^*) \beta (e^{\gamma t^*} - 1)}{\gamma e^{\gamma t^*} (P_1(t^*) \beta_1' + P_2(t^*) \beta_2')} \tag{27}$$

As mentioned before, the ratio of lysogens when steady-state is reached is

$$\frac{L_1}{L_2} = \frac{f_1 P_1(t^*)}{f_2 P_2(t^*)},$$

and this can be taken as the payoff Z for phage 1, with strategy f_1, when competing against phage 2, with strategy f_2. This is a monotonic function of the payoff used in the main text, $Z_{\text{maintext}} = (L_1 - L_2)/(L_1 + L_2)$, so minimax strategies calculated from this will be the same as calculated using Z_{maintext}. So, the payoff for phage 1 as a function of the two competing

strategies is:

$$Z(f_1, f_2) = \frac{f_1}{f_2} e^{\frac{\eta B_0(0)\beta}{\gamma}(f_2 - f_1)\left(e^{\gamma t^*} - 1\right)} \tag{28}$$

To calculate the minimax strategy, we first maximize Z with respect to f_1:

$$\frac{\partial Z}{\partial f_1} = 0 \tag{29}$$

$$\Rightarrow \frac{1}{f_2} e^{\frac{\eta B_0(0)\beta}{\gamma}(f_2 - f_1)\left(e^{\gamma t^*} - 1\right)} + \frac{f_1}{f_2} e^{\frac{\eta B_0(0)\beta}{\gamma}(f_2 - f_1)\left(e^{\gamma t^*} - 1\right)} \frac{\eta B_0(0)\beta}{\gamma} \left[\gamma e^{\gamma t^*} \frac{\partial t^*}{\partial f_1}(f_2 - f_1) - e^{\gamma t^*} + 1\right] = 0 \tag{30}$$

$$\Rightarrow \frac{1}{f_1} = \frac{\eta B_0(0)\beta}{\gamma} \left[\gamma e^{\gamma t^*} \frac{\partial t^*}{\partial f_1}(f_1 - f_2) + e^{\gamma t^*} - 1\right] = 0 \tag{31}$$

$$\Rightarrow \frac{1}{f_1} = \frac{\eta B_0(0)\beta}{\gamma}(e^{\gamma t^*} - 1) \left[\frac{(f_1 - f_2)P_1(t^*)\beta}{P_1(t^*)\beta_1' + P_2(t^*)\beta_2'} + 1\right] \tag{32}$$

$$\Rightarrow \frac{1}{f_1} = \frac{\eta B_0(0)\beta\beta_2'(P_1(t^*) + P_2(t^*))}{\gamma(P_1(t^*)\beta_1' + P_2(t^*)\beta_2')}(e^{\gamma t^*} - 1) \tag{33}$$

Thus, for each value of f_2, the value of f_1 that maximizes $Z(f_1, f_2)$ is given by $f_1 = f_1^*(f_2)$ where f_1^* satisfies the above equation (it is tedious, but straightforward, to show using the value of the second derivative that we are looking at a true maximum, so we have not included that calculation here). The minimax strategy for phage 2 is then the value of f_2 that minimizes $Z(f_1^*(f_2), f_2)$. The most straightforward way to calculate this is from the equation:

$$\frac{\partial Z(f_1^*(f_2), f_2)}{\partial f_2} = \frac{\partial}{\partial f_2}\left[\frac{f_1^*(f_2)}{f_2} e^{\frac{\eta B_0(0)\beta}{\gamma}(f_2 - f_1^*(f_2))\left(e^{\gamma t^*(f_1^*(f_2), f_2)} - 1\right)}\right] = 0 \tag{34}$$

$$\Rightarrow \frac{\partial}{\partial f_2}\left[\frac{f_1^*(f_2)}{f_2} e^{\frac{\eta B_0(0)\beta}{\gamma}(f_2 - f_1^*(f_2))\left(e^{\gamma t^*(f_1^*(f_2), f_2)} - 1\right)}\right] = 0 \tag{35}$$

However, this is also quite tedious, so we will take a shortcut by using the fact that this is a completely symmetric game, so the minimax strategy must be the same for both players (this symmetry is also why the seemingly crude calculation in the main text, of maximising $f_1 P_1(t^*)$, gives the same result). That is, the minimax strategy must simultaneously satisfy

$f_1 = f_2(\equiv f_{opt})$ and eq (33). Combining these two we get:

$$\frac{1}{f_{opt}} = \frac{\eta B_0(0)\beta}{\gamma}(e^{\gamma t^*(f_{opt},f_{opt})} - 1) \tag{36}$$

The right hand side of the above equation can be further simplified by noting that t^* marked the switch from the buildup to the crash stage, i.e.,

$$\eta P_{tot}(t^*) = \gamma \tag{37}$$

$$\Rightarrow \eta P_1(0) e^{\frac{\eta \beta_1' B_0(0)}{\gamma}(e^{\gamma t^*}-1)} + \eta P_2(0) e^{\frac{\eta \beta_2' B_0(0)}{\gamma}(e^{\gamma t^*}-1)} = \gamma \tag{38}$$

When $f_1 = f_2 \equiv f_{opt}$ and $P_1(0) = P_2(0)(\equiv P(0))$,

$$\Rightarrow 2\eta P(0) e^{\frac{\eta \beta' B_0(0)}{\gamma}(e^{\gamma t^*(f_{opt},f_{opt})}-1)} = \gamma \tag{39}$$

$$\Rightarrow \frac{\eta B_0(0)\beta}{\gamma}(e^{\gamma t^*(f_{opt},f_{opt})} - 1) = \frac{\beta}{\beta'} \ln \frac{\gamma}{2\eta P(0)} \tag{40}$$

$$\Rightarrow \frac{\eta B_0(0)\beta}{\gamma}(e^{\gamma t^*(f_{opt},f_{opt})} - 1) = \frac{\beta}{\beta(1-f_{opt})-1} \ln \frac{\gamma}{2\eta P(0)} \tag{41}$$

$$\Rightarrow \frac{1}{f_{opt}} = \frac{\beta}{\beta(1-f_{opt})-1} \ln \frac{\gamma}{2\eta P(0)} \tag{42}$$

$$\Rightarrow f_{opt} = \frac{1 - \frac{1}{\beta}}{1 + \ln \frac{\gamma}{2\eta P(0)}} \tag{43}$$

Figure S4 compares the formula (43) with the results of simulations of the model. The formula (black curve) matches the simulations with very large δ (red curve) reasonably well; it starts to break down for particularly large η, $B_0(0)$, $P_i(0)$, and small γ, β. For a lower, more biologically reasonable value of δ (blue curve) the formula underestimates the simulation value by about Δf_{opt}=0.03, but reproduces the general trends and the fact that f_{opt} changes relatively little even when the parameters are changed by several orders of magnitude.

1.5 Exploring strategies that depend on multiplicity of infection

In addition to the evolutionary game described in the main text in section "Methods: Including multiple infections in the model", we also calculated the payoff matrix and determined the minimax strategy, as outlined elsewhere, for various restricted sets of strategies that depend on the multiplicity of infection:

1. We fixed $f_i(1) = 0$ and $f_i(3) = 1$ (for $i = \{1, 2\}$) and explored all values of $f_1(2)$ and $f_2(2)$ ("all values" means 0, 0.01, 0.02, ..., 0.99, 1)

2. We fixed $f_i(1) = 0$ and $f_i(2) = 1$ (for $i = \{1, 2\}$) and explored all values of $f_1(3)$ and $f_2(3)$

3. We fixed $f_i(2) = 1$ and $f_i(3) = 1$ (for $i = \{1, 2\}$) and explored all values of $f_1(1)$ and $f_2(1)$

4. We fixed $f_i(1) = 0$ and explored all values of $f_1(3)$ and $f_2(3)$, with the constraint that $f_1(2) = f_1(3)$ and $f_2(2) = f_2(3)$.

For the default parameter values, in all these cases, the optimal minimax strategy was $f(1) \approx 0$, $f(2) \approx f(3) \approx 1$.

We hypothesize that this strategy does well because, amongst the strategies we have examined, it is best able to "sense" the onset of the crash period and switch from a predominantly lytic strategy to a predominantly lysogenic strategy at that point, for a wide range of parameter values. More quantitatively, we predict that other strategies make more "errors", defined as lytic events in the build-up phase and lysogenic events in the crash and post-crash phase. From the dynamics of $B_{i,m}(t)$, we can calculate this error for each phage variant:

$$\Delta_i = \int_0^{t^*} \frac{\sum_{m=1}^3 f_i(m) B_{i,m}}{\sum_{m=1}^3 \left[f_i(m) B_{i,m} + (1 - f_i(m)) B_{i,m}\right]} + \int_{t^*}^{\infty} \frac{\sum_{m=1}^3 (1 - f_i(m)) B_{i,m}}{\sum_{m=1}^3 \left[f_i(m) B_{i,m} + (1 - f_i(m)) B_{i,m}\right]}, \quad (44)$$

for $i = \{1, 2\}$. We ran more than 25000 games with player 1 having the strategy $f(1) = 0, f(2) = f(3) = 1$ versus player 2 having various strategies, also covering various values of other parameters. Figure S5 plots $\Delta_2 - \Delta_1$ (a positive value of this indicates that strategy $f(1) = 0, f(2) = f(3) = 1$ made less such errors than the strategy it was competing against) as a function of payoff margin. 25000 of these data points are games where the competing strategy was chosen randomly, with parameters also chosen randomly from the range given in Column 3 of Table 1, while 6 (which are labeled) were against some selected strategies, for default parameter values. Note that whenever the strategy $f(1) = 0, f(2) = f(3) = 1$ wins (and it wins 25004 of these games), it makes fewer errors (as defined above) than the opposing strategy. In the two cases where $f(1) = 0; f(2) = f(3) = 1$ is beaten, the winning strategies were (i)$f(1) = 0; f(2) = 0.58; f(3) = 0.99$ and (ii) $f(1) = 0; f(2) = 0.58; f(3) = 0.99$. Incidentally, for these two parameter sets, the optimal winning strategy obtained from the evolutionary game is $f(1) = f(2) = 0; f(3) = 1$.

1.6 Spatial model

We set up a two-dimensional square grid where each site can contain at most one bacterium. The carrying capacity is thus equal to the number of sites in the grid. Each bacterium may be in one of the following states: uninfected, infected by phage variant 1, infected by phage variant 2, lysogen of variant 1, lysogen of variant 2. Bacteria are not allowed to move on the grid. For each bacterium, a counter keeps track of the time since the previous division, and when this time crosses a threshold value (randomly chosen from the uniform distribution on the interval $[0.9\tau \;\; 1.1\tau]$) it divides, with the new cell placed randomly in an adjacent empty site, if such exists. The division counters for both cells are then reset to zero. If no new site exists then no division occurs but the cell's counter is reset. This growth rule produces approximately logistic growth of bacterial colonies with growth rate $\gamma = \ln 2/\tau$.

For simplicity, we model the phages indirectly as follows: when an infected cell bursts, then

all adjacent uninfected bacteria immediately become infected. This corresponds to the limit where η, the phage infectivity, is very large so the timescales of phage infection are much faster than other timescales in the system. When there are no uninfected bacteria adjacent to a burst, the phages are lost, which corresponds to assuming that the typical length scale associated with phage diffusion in its lifetime is relatively short, so phages are unlikely to infect bacteria that are not nearest neighbours of the burst. The transition from being infected to either bursting or becoming a lysogen is modeled as a Poisson process, i.e., there is a fixed probability per unit time δf for an infected cell to become a lysogen, and $\delta(1-f)$ for it to burst, where f is the lysogeny propensity of the phage variant corresponding to that particular infected cell.

When an infected cell is adjacent to a burst, we study two versions of the model: first, the adjacent infected cell remains unaffected; second, we keep track of multiple infections, i.e., the multiplicity of infection of an infected cell increases (upto a maximum of 3; beyond 4, the lysogeny propensity is set as 1) when it is adjacent to a burst. The lysogeny propensity in this latter case is allowed to vary with the multiplicity of infection, but all other parameters are unaffected.

To determine optimal lysogeny propensities for these spatial models we use exactly the same approach as for the well-mixed models. Figure S6 shows that, similar to the well-mixed model, when we compare fixed lysogeny propensity strategies, the optimal strategy is $f_{opt} \approx 0.1$ and is quite robust to large changes in the parameter values τ and δ and initial conditions. Similarly, when we simulate 5000 iterations of the spatial model with multiplicity of infection, in each iteration replacing the losing strategy by a mutated version of the winning strategy (just as in section "Phages that count multiplicity of infection compete better" of Results in the main text), we obtain a switch-like winning strategy, albeit with the switch occurring between MOI = 2 and MOI = 3 (see Figure S7).

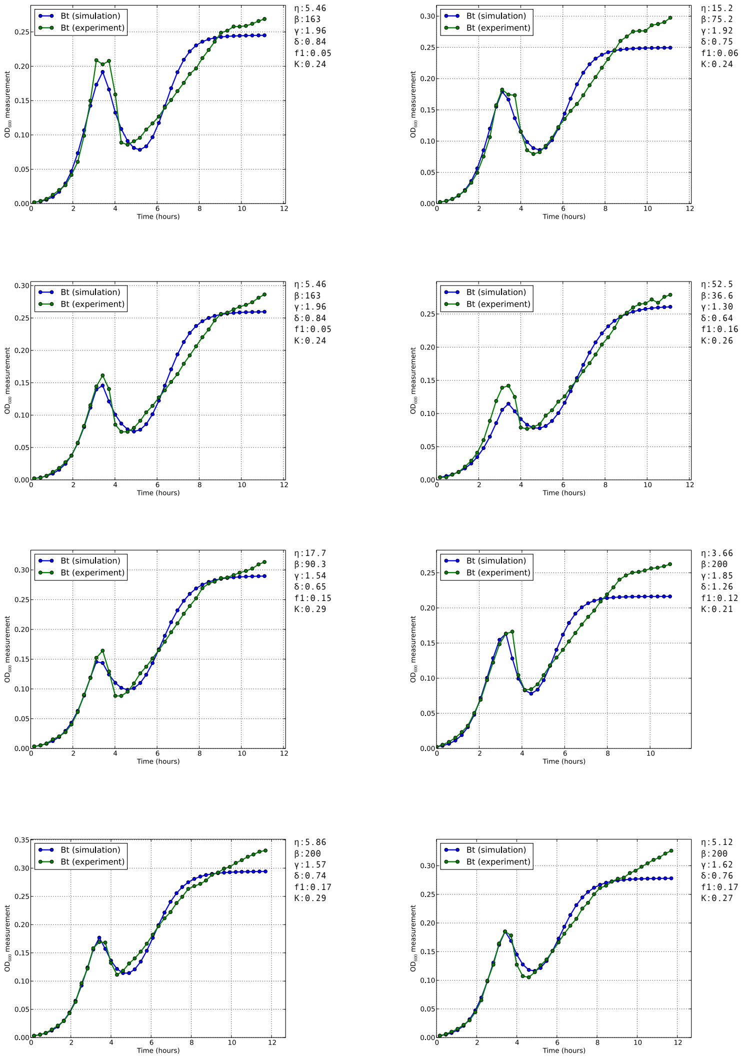

S 1: *Fitting our model (eqs. 1–4) to data from 8 experiments where wild-type E. coli K12 populations were infected by λ phage [Maynard et al., 2010]. Green curves show OD600 measurements of total bacterial density as a function of time, and blue curves show the best fit of eqs. (1)–(4) obtained as described in section 1.2. The parameters corresponding to each fit are displayed to the right of each panel.*

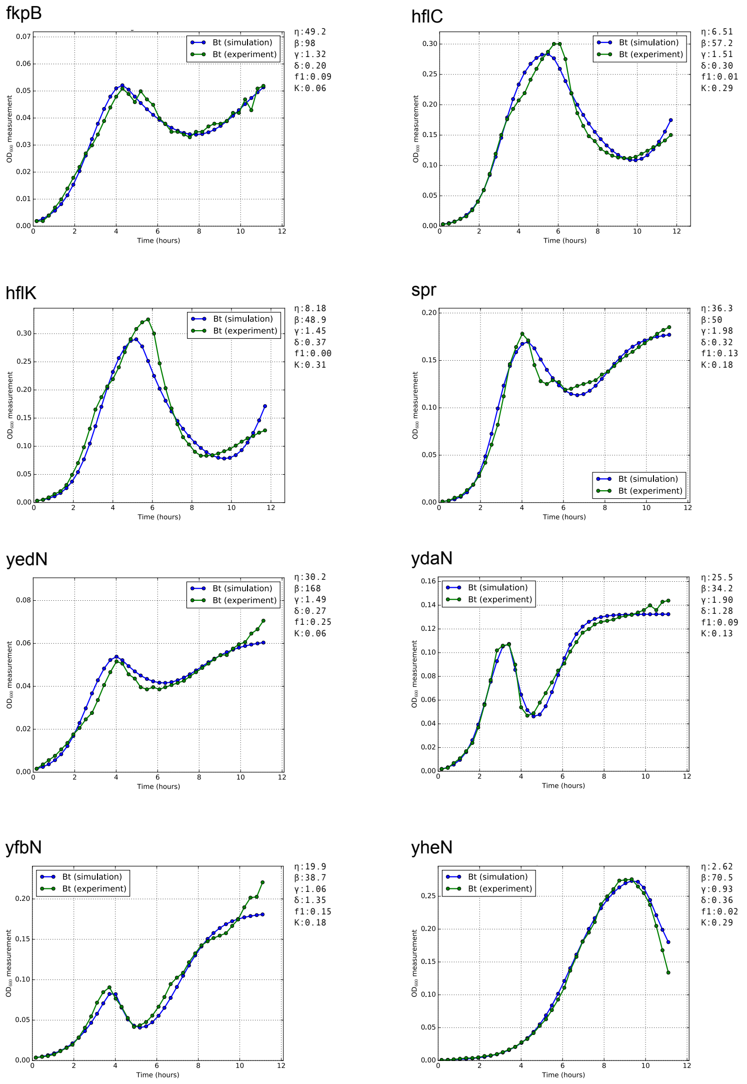

S 2: *Fitting our model (eqs. 1–4) to data from 8 experiments where various E. coli mutant populations (see top left label on each panel) were infected by λ phage [Maynard et al., 2010]. Green curves show OD600 measurements of total bacterial density as a function of time, and blue curves show the best fit of eqs. (1)–(4) obtained as described in section 1.2. The parameters corresponding to each fit are displayed to the right of each panel.*

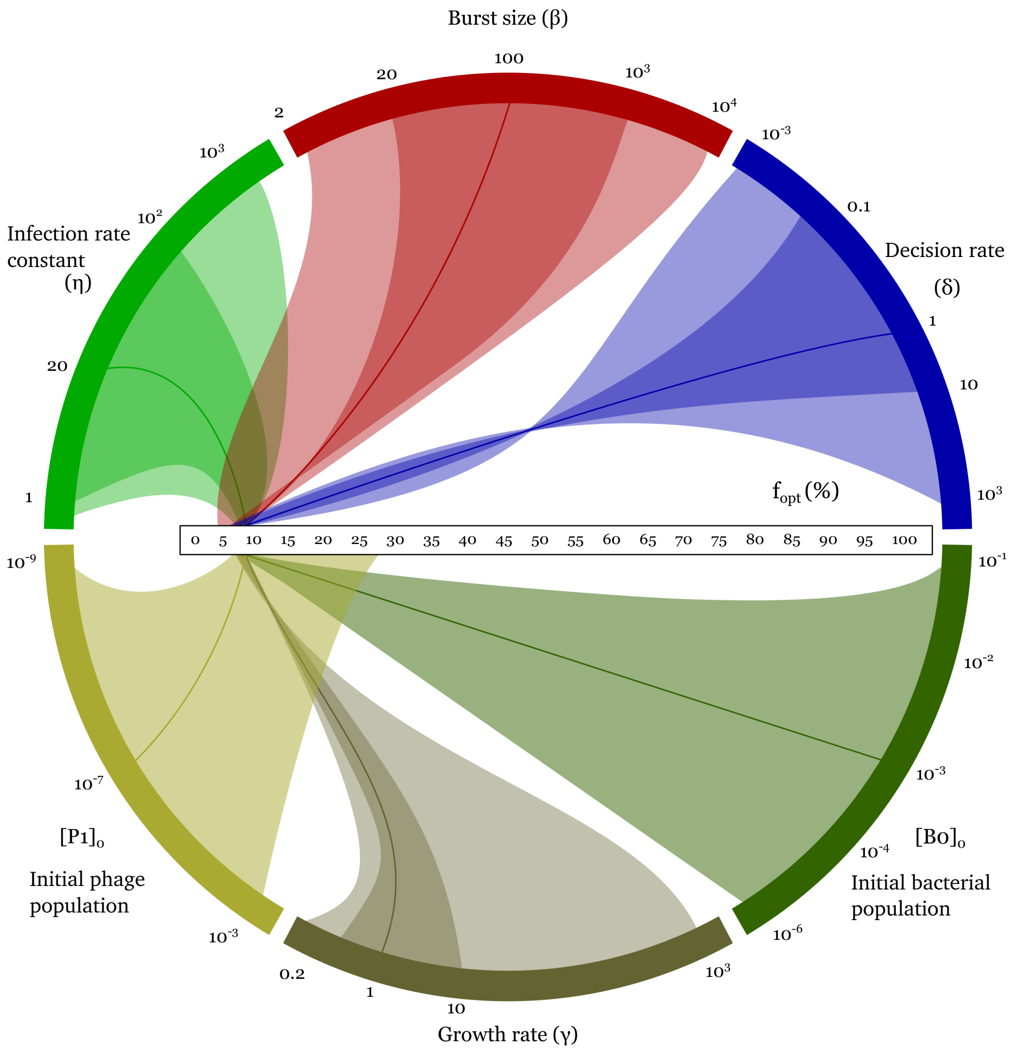

S 3: *Variation of the optimal (minimax) lysogeny propensity, f_{opt}, as parameters are varied one by one over the range shown in column 4 of Table 1, for the model that allows cross-infections (eqs. 9–14). Similar to Figure 4 of the main text, this figure illustrates the robustness of f_{opt} over a wide range of parameter values and initial conditions. Every coloured arc corresponds to one parameter. The horizontal scale in the middle is the range of possible f_{opt} values, from 0-100%. The solid darker curve connecting each parameter arc to the f_{opt} scale corresponds to the default parameter set used in Figures 2 and 3 in the main text. The darker and lighter areas connecting the arcs to the f_{opt} scale mark, respectively, the biologically reasonable range and the full parameter range explored, i.e., columns 3 and 4 of Table 1. The twists indicate decreasing trends for increasing parameter values.*

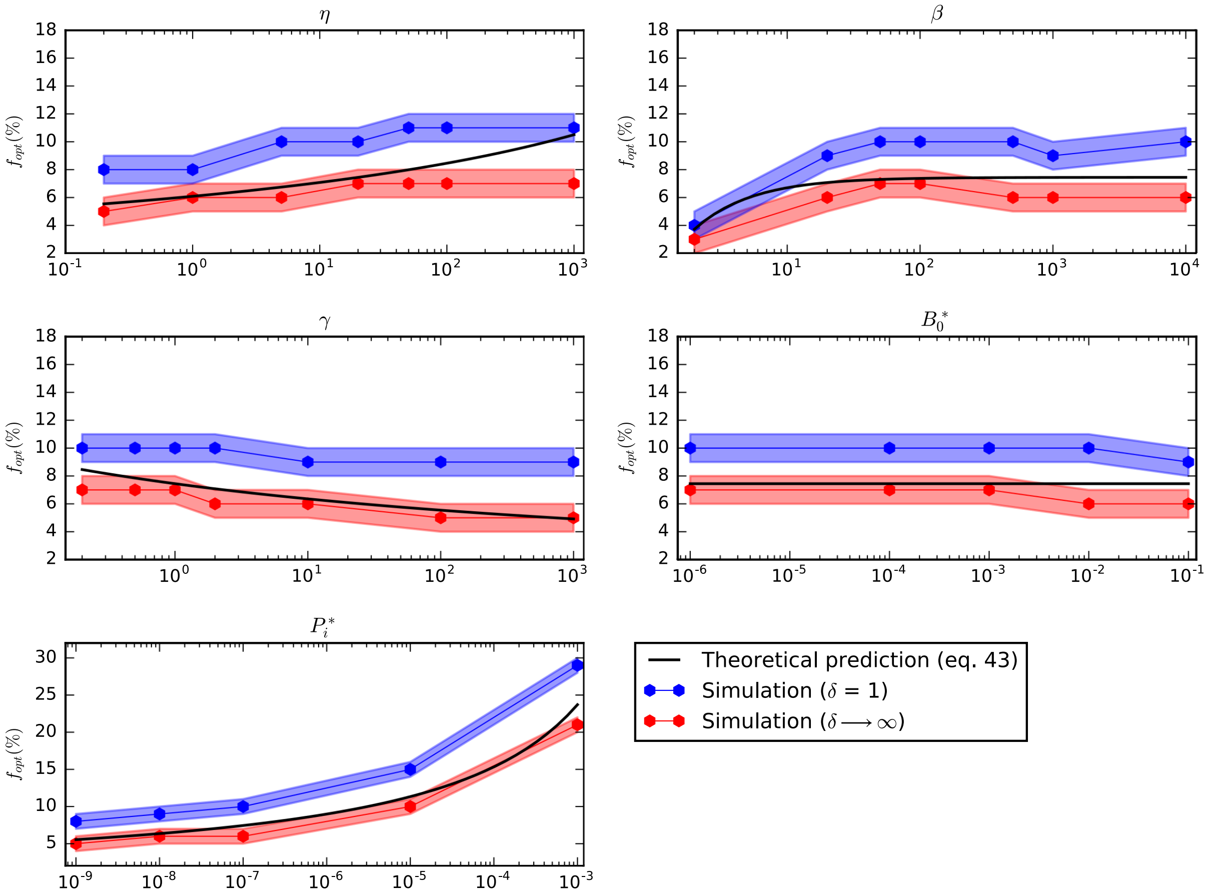

S 4: *Dependence of f_{opt} on individual parameters, for the model described by eqs. (1)–(4). The blue curves are an alternate visualisation of the same data shown in Figure 4 in the main text, with the decision rate at $\delta = 1$ hr^{-1}. In addition, the black curve plots the theoretical prediction, eq. (43), and the red curve shows the results of simulations when δ is assumed to be infinitely large. The shaded coloured areas correspond to the"least count" of $\Delta f = \pm 0.01$ in our measurements of the lysogeny propensity, f.*

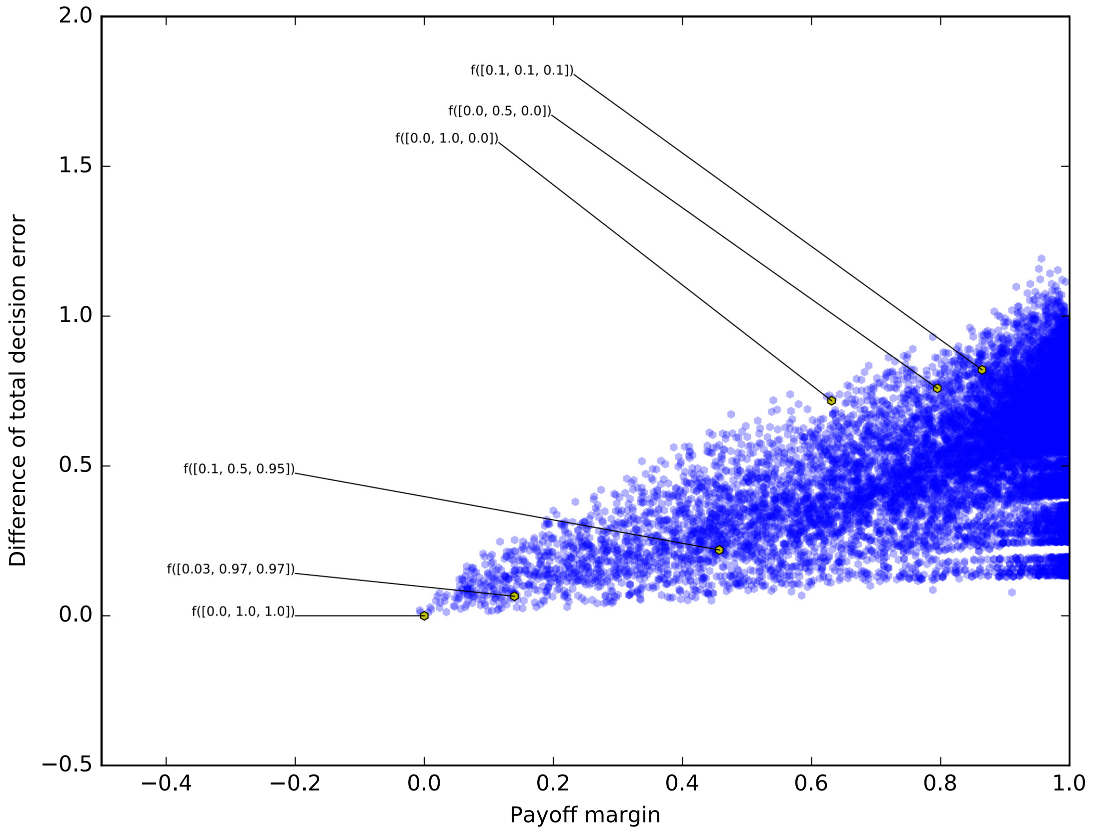

S 5: *The difference in errors made by various strategies playing against* $f(1) = 0; f(2) = f(3) = 1$. *The dots show* $\Delta_2 - \Delta_1$ *(with the "error"* Δ_i *defined by eq. 44) for 25006 simulations of the model that allows multiplictity of infection, eqs. (5)–(9) in the main text, where player 1 has strategy* $f(1) = 0; f(2) = f(3) = 1$ *and player 2 has either randomly chosen strategies (blue dots) or the labelled strategies (yellow dots). In all cases,* $\Delta_2 - \Delta_1 \geq 0$. *The parameters for each of these 25006 simulations were chosen randomly from the range specified by column 3 in Table 1. In only 2 simulations did player 1 lose, in both cases to the strategies (i)* $f(1) = 0; f(2) = 0.58; f(3) = 0.99$ *and (ii)* $f(1) = 0; f(2) = 0.58; f(3) = 0.99$*; in these simulations the parameter values were (i)* $K = 1$ *,*η*=50,* β*=20,* γ*=10,* δ*=0.5,* $B_0(0)$=10^{-3}, $P_i(0)$=10^{-9}. *and (ii)* $K = 1$, η*=50,* β*=1000,* γ*=2,* δ*=0.2,* $B_0(0)$=10^{-3}, $P_i(0)$=10^{-7}. *For both sets of parameter values, the optimal winning strategy obtained from the evolutionary game is* $f(1) = f(2) = 0; f(3) = 1$.

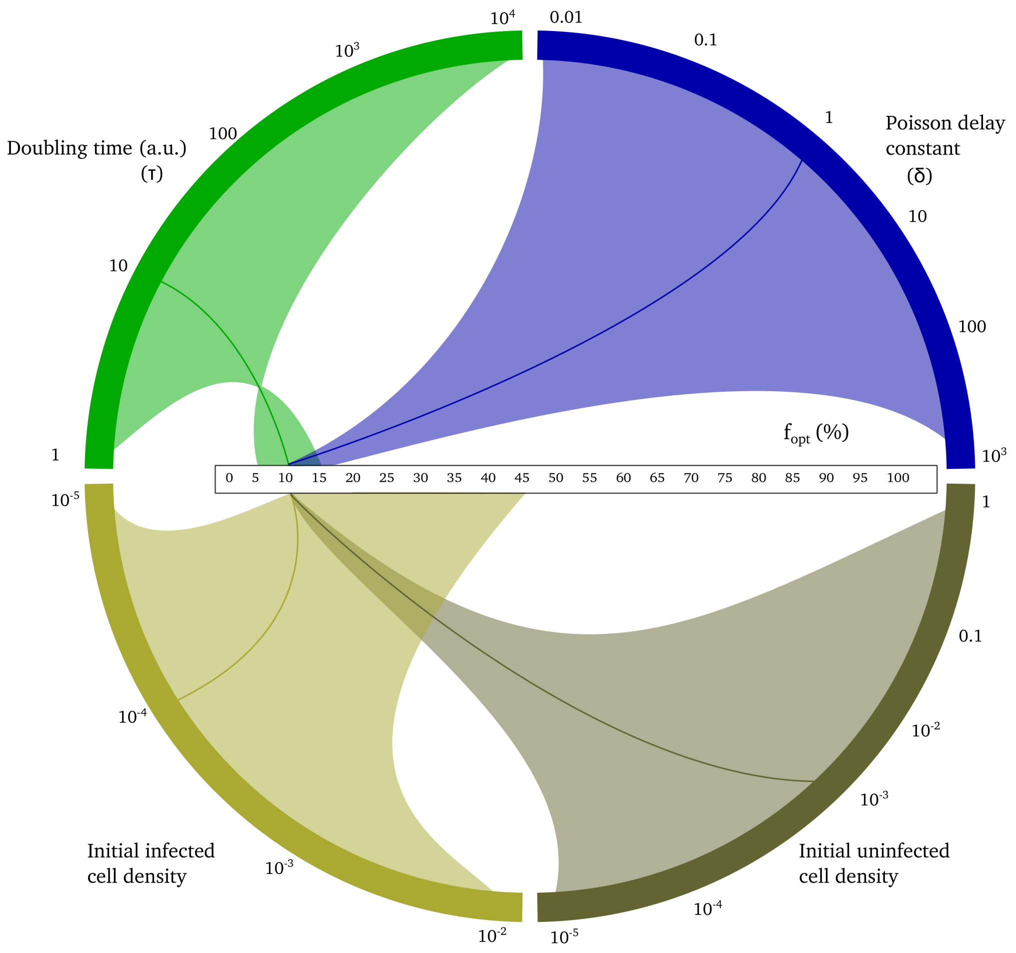

S 6: *Variation of the optimal (minimax) lysogeny propensity, f_{opt}, as parameters are varied one by one, for the spatial model described in section 1.6. As in Figure 4 in the main text, and Figure S3, this figure demonstrates the robustness of f_{opt} over a wide range of parameter values and initial conditions.*

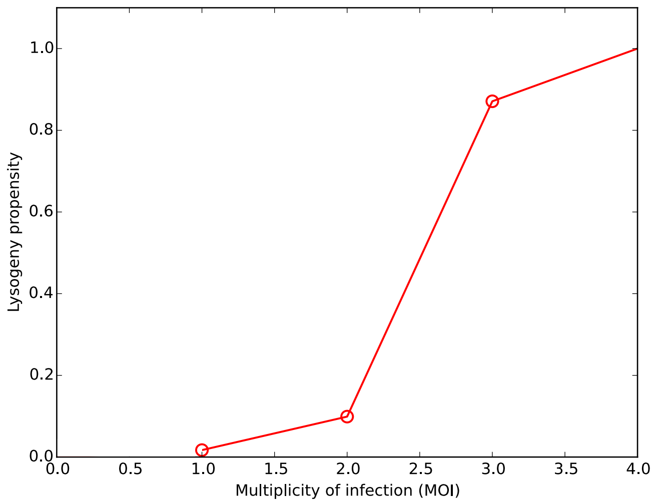

S 7: *The winning strategy $f(MOI)$, for the spatial model with multiple infections, obtained after 5000 rounds of replacing the losing strategy by a mutated version of the winning strategy, similar to the procedure for producing Figure 5 in the main text.*

References

[Maynard et al., 2010] Maynard, N.D., Birch, E.W., Sanghvi, J.C., Chen, L., Gutschow, M.V., Covert, M.W. (2010). A forward-genetic screen and dynamic analysis of lambda phage host-dependencies reveals an extensive interaction network and a new anti-viral strategy. PLoS Genet **6**:e1001017. doi: 10.1371/journal.pgen.1001017
